# A novel ZnS-CdS nanocomposite as a visible active photocatalyst for degradation of synthetic and real wastewaters

**DOI:** 10.1038/s41598-023-28725-7

**Published:** 2023-02-07

**Authors:** Minoo Khodamorady, Kiumars Bahrami

**Affiliations:** 1 Department of Organic Chemistry, Faculty of Chemistry, Razi University, Kermanshah, 67144-14971 Iran; 2 Nanoscience and Nanotechnology Research Center (NNRC), Razi University, Kermanshah, 67144-14971 Iran

**Keywords:** Environmental sciences, Environmental chemistry, Photochemistry, Photocatalysis

## Abstract

In this study, new magnetic nanocomposites with shell core structure with different molar ratios of ZnS-CdS were synthesized and their photocatalytic activity in dye removal from synthetic and real effluents in the presence of mercury high pressure lamp as a visible light source was investigated. Optimal photocatalyst with molar ratio of ZnS-CdS 0.25:0.75 showed the best performance in dye removal. Based on the particle distribution histogram of Fe_3_O_4_@BNPs@ZnS-CdS (ZnS/CdS: 0.25:0.75), particles with 60–100 nm have the highest abundance. According to the DRS results, hybridization of zinc sulfide with cadmium sulfide reduced the gap and as a result, light absorption was successfully extended to the visible area. The PL results confirm that the optimal photocatalyst (Fe_3_O_4_@BNPs@ZnS-CdS) has the lowest electron–hole recombination compared to Fe_3_O_4_@BNPs@ZnS and Fe_3_O_4_@BNPs@CdS. It should be noted that according to the DLS results, the charge on the optical photocomposite surface is negative at all acidic, alkaline and neutral pHs. One of the significant advantages in this study is the use of high-pressure mercury lamps as a light source, so that these lamps are very economical in terms of economy and also have a long life and excellent efficiency. The optimal photocatalyst not only showed excellent photocatalytic activity for the removal of methylene blue (96.6%) and methyl orange (70.9%) but also for the dye removal of textile effluents (Benton 98.5% and dark olive 100%). Introduced magnetic heterostructures are suitable options for dye removal from textile and spinning wastewaters.

## Introduction

Today, along with industrialization, population growth and increasing unlimited human activities, water, soil and air pollution has also increased and various pollutants such as (dyes, drugs and pesticides) enter the water, which has a direct impact on Environment, human health, animals, birds, underwater creatures^1–4^.

Air pollution leads to asthma and respiratory diseases. Soil pollution affects agriculture and causes stomach diseases^5,6^. Today, the issue of water pollution is the most serious issue because industries such as textiles, printing, dyeing, spinning, leather, etc. discharge large amounts of wastewater containing dyes and toxic substances into the environment, which affects the quality and health of water^7–9^. Dyes are classified into alkaline, acidic, neutral, azo, and radioactive dyes based on their charge and application.

Dyes are one of the most dangerous organic pollutants in industrial effluents, especially textile effluents, which have high toxicity and indestructible structure that cause cancer and genetic mutations, dermatitis, allergies, skin irritation in humans. Dyes may disrupt the process of photosynthesis in aquatic ecosystems because they prevent light from penetrating into the water^10,11^. The discharged effluent of the textile industry is about 100 tons of colored effluent per year, which leads to water pollution^12,13^.

Since a significant percentage (70%) of diseases originate from polluted water^14^, and also due to population growth and declining groundwater and drought, water treatment is the best solution to solve the crisis of shortage of safe water.

Generally, water treatment methods include three basic categories of chemical, physical and biological methods^15^.

In recent decades, different techniques have been used for the removal of organic pollutants and water treatment, such as membrane separation, use of various adsorbents, use of semiconductors and photocatalysts, some of which, in addition to being complex, are costly^1,2^. Among the reported techniques, photocatalytic degradation is one of the best methods for water treatment and removal of organic pollutants in wastewater.

So far, semiconductors such as ZnO, ZnS, CdS, TiO_2_, NiO, Fe_2_O_3_, SnO_2_, CeO, Ag_2_O have been used^2,16–19^. The main problem of most semiconductors is the large band gap, which leads to inactivity of the semiconductor in the visible area. Metal sulfides can receive directly visible light due to their shorter band gap than metal oxides, and are therefore superior to metal oxides^20^. Zinc sulfide with a band gap of 3.72–3.77 eV has an absorption edge lower than 340 nm, which is active only in the UV region. To increase the activity of semiconductors, they are doping with different percentages of metal oxides or sulfides, various transition metals such as Ni, Fe, Mn and Co and non-metals like S, N, F and O^21–24^. Hybridization reduces band gap and allows the photocatalyst to operate in the visible region.

CdS is another semiconductor that has been widely used in the photocatalytic degradation of organic pollutants and water splitting. CdS is an n-type semiconductor with a band gap of 2.42 eV that is able to absorb good light in the visible light region^25,26^.

Numerous studies have been reported on the synthesis and application of CdS-ZnS hybrid nanostructures. For example, Reddy et al.^27^ succeeded in synthesizing CdS-ZnS nanoparticles with a core–shell structure and used it for photocatalytic removal of methyl orange dye. In another study, Amiri's research group used CdS-ZnS nanoparticles to investigate the removal of heavy metals^28^.

Nanotechnology today makes it possible to design and fabricate recyclable hybrid nanocomposites with the efficiency and selectivity of homogeneous catalysts^29^.

One of the materials that is widely used in the synthesis of nanocatalysts today is iron oxide nanoparticles, which have been considered due to their high biocompatibility. Fe_3_O_4_ has superior magnetic properties, good electronic conductivity and high biocompatibility, has attracted more attention than other iron oxides^30^. The magnetic properties of Fe_3_O_4_ can be related to its crystal structure. Magnetite nanoparticles (Fe_3_O_4_) (NPs) have many applications in the fields of materials science, physics and chemistry due to their noteworthy properties like being semi-metallic, ferromagnetic and environmentally friendly^31–33^.

Another inexpensive, stable and recyclable solid nanomaterial whose surface is easily functionalized is boehmite. Boehmite is AlOOH, which is a hydrophilic substrate due to its surface being covered with a large number of hydroxy groups, and its surface is easily functionalized^34,35^. The hybridization of iron nanoparticles with boehmite nanoparticles, in addition to improving the chemical and mechanical properties of nanoparticles, also increases the hydrophilic property of nanoparticles. As a benefit, the hydrophilicity of the support in photocatalytic processes improves the photocatalytic performance.

In this study, the technique of coupling ZnS with CdS was used to reduce the gap band and reduce electron–hole recombination and increase the ability to absorb visible light. Modified magnetic nanocomposites with different molar ratios of ZnS-CdS were synthesized. The photocatalytic activity of these nanocomposites was compared with Fe_3_O_4_@BNPs@ ZnS and Fe_3_O_4_@BNPs@ CdS to remove methylene blue and methyl orange dyes from synthetic effluents as well as to dye removal of textile and west carton effluents.

Based on the results, the optimal photocatalyst (Fe_3_O_4_@BNPs@ ZnS-CdS) (0.25: 0.75) showed the best performance for dye removal in the presence of high pressure mercury lamp as the visible light source.

## Experimental

### Materials and methods

Materials used in this study are: hydrated aluminum nitrate (Al(NO_3_)_3_.9H_2_O), iron sulfate (FeSO_4_), iron chloride (FeCl_3_), sodium hydroxide (NaOH), sodium sulfide (Na_2_S), cadmium acetate (Cd (OAc)_2_, zinc acetate (Zn(OAc)_2_), benzoquinone, ammonium oxalate, silver nitrate, tert-butanol, methyl orange dye (C_14_H_14_N_3_NaO_3_S, MW: 327.34 g mol^−1^), methylene blue dye (C_16_H_18_ClN_3_S, M = 319.85 g mol^−1^), ethanol, etc., all of which were purchased from Merck. The visible light source used in this study is high pressure mercury lamps. In order to check the light intensity of a mercury lamp, a device called lux meter was used and the fluctuation in light intensity was observed. The lowest intensity for visible light is 95 lumens per minute and the highest intensity is 115 lumens per minute, and the average light intensity of the source is 105 lumens per minute. FT‐IR spectra were recorded on a Shimadzu IR-470 spectrometer. TGA was carried out with a STA504 in the temperature range of 25–800 °C at a heating rate of 10 °C min^−1^. FESEM and EDX measurements were performed by a TESCAN‐MIRA3. XRD pattern was obtained by using a JEOL-JDX-8030 (30 kV, 20 mA). Zeta potential was recorded on SZ-100z Dynamic Light Scattering & Zeta potential analyze (Horiba Jobin)*.* Magnetic property of the photocatalyst was obtained by VSM + FORC vibrating sample magnetometer. The DRS spectrum of Fe_3_O_4_@BNPs@ZnS-CdS was recorded by Avaspec-2048-TEC. Also, photoluminescence spectra were gained by Perkin Elmer LS55. Degradation of MB and MO was monitored by UV–visible absorption spectroscopy (UV-1650PC SHIMADZU, Columbia, MD, USA).

### Preparation of Fe_3_O_4_@BNPs

First, a mixture of FeCl_3_ (1.55 g), FeSO_4_ (1.05 g) and 5% NaOH solution is mixed and placed under N_2_ gas for 2 h at 90 °C under stirrer condition. After synthesis, the Fe_3_O_4_ NPs are separated by an external magnet and washed with water and ethanol. In the next step, a solution of Al(NO_3_)_3_ is added to Fe_3_O_4_ and then the solution of NaOH is added drop by drop and the mixture is placed in an ultrasonic bath.

### Synthesis of Fe_3_O_4_@BNPs@ZnS

Zinc acetate solution was added to Fe_3_O_4_@BNPs by sonication and the mixture was refluxed at room temperature for 1 h under intense stirrer. A solution of Na_2_S was then added and the mixture was refluxed again at 60 °C for 2 h. The prepared Fe_3_O_4_@BNPs@ZnS nanoparticles were cooled, separated by an external magnet and dried after rinsing with water at 60 °C.

### Synthesis of Fe_3_O_4_@BNPs@CdS

For the modification of magnetic boehmite with Cd(OAc)_2_, a solution of cadmium acetate was added to Fe_3_O_4_@BNPs under sonication, and the resulting mixture was refluxed at room temperature for 60 min. A solution of sodium sulfide was then added to the mixture and the mixture was refluxed again at 60 °C. Finally, the Fe_3_O_4_@BNPs@CdS was washed with water after separation with a strong external magnet and dried overnight at 60 °C.

### Synthesis of Fe_3_O_4_@BNPs@ ZnS-CdS

Separate aqueous solutions of Zn(OAc)_2_ and Cd(OAc)_2_ were added to Fe_3_O_4_@BNPs (0.5 gr) under sonication, and the mixture was refluxed at rt for 1 h. After that, the mixture was well stirred, a solution of Na_2_S was added and the mixture was refluxed at 60 °C. The synthesized Fe_3_O_4_@BNPs@ ZnS-CdS nanocomposite was separated after cooling, separated by a strong external magnet, washed with water and dried in an oven at 60 °C.

### Dye removal experiments in the presence of Fe_3_O_4_@BNPs@ ZnS-CdS

The performance of photocatalysts in dye removal from synthetic effluents of MB and MO at concentrations of 10, 15 and 20 ppm was investigated. Then, important parameters such as the amount of photocatalyst, dye concentration and pH of the environment were surveyed. After the studies, the best photocatalyst (Fe_3_O_4_@BNPs@ ZnS-CdS) was chosen.

The photocatalyst’s activity in removing dye from Kashan textile effluent, which includes sausage, benton and dark olive effluents with concentrations of 15–20 ppm, as well as Kermanshah west carton company effluent (dark olive effluent) were examined.

## Result and discussion

Three photocatalysts were prepared with different molar ratios from ZnS-CdS. The performance of these photocatalysts was evaluated with Fe_3_O_4_@BNPs@CdS and Fe_3_O_4_@BNPs@ZnS photocatalysts in the visible light region. Based on the results, it was found that the optimal photocatalyst in the visible light region is Fe_3_O_4_@BNPs@ZnS-CdS with a molar ratio of ZnS:CdS = 0.25:0.75.

**Figure 1:**
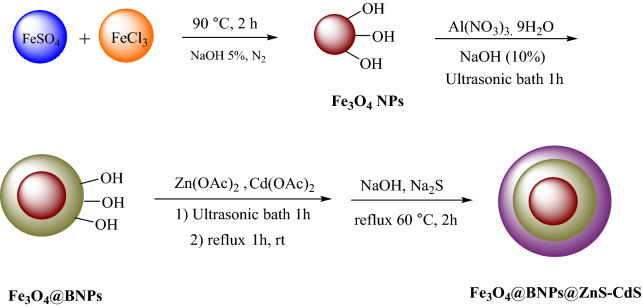
Photocatalyst synthesis steps.

Several techniques like FTIR, XRD, FESEM, EDX, TGA, PL, DRS, BET, VSM, zeta potential were accomplished to corroborate the structure and evaluate the attributes of the best photocatalyst. The schematic steps of the photocatalyst synthesis are illustrated in Fig. 1.

### Characterization of the Fe_3_O_4_@BNPs@ZnS-CdS

**Figure 2:**
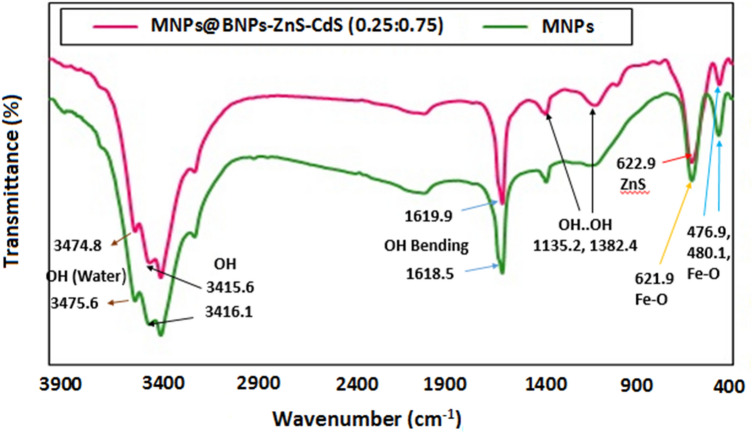
FT-IR spectra of MNPs and Fe_3_O_4_@BNPs@ZnS-CdS.

Infrared spectra of MNPs and photocatalyst are shown in Fig. 2. As Fig. 2 shows, the vibrational frequencies appearing in region 480.1 cm^−1^ are related to the octahedral Fe–O tensile vibration and 621.9 cm^−1^ are related to the Fe–O tetrahedral tensile vibration. The tensile vibration that appeared in 1133.6 cm^−1^ belongs to the OH of water molecules. The peak that appeared in 1618.5 cm^−1^ also belongs to the bending vibration of the hydroxyl group. The vibrations appearing in 3416.1 and 3475.6 cm^−1^ are related to hydroxy groups on the surface of iron nanoparticles and adsorbed water molecules^36^.

In the FT-IR spectrum of Fe_3_O_4_@BNPs@ ZnS-CdS, the vibrations appearing at 476.9 and 622.9 cm^−1^ are related to Fe–O and Zn–S, respectively^37,38^. The peak that appeared in 1619.9 cm^−1^ is assigned to the bending vibration of the hydroxyl group^20^. The vibrations appearing in 3415.6 and 3474.8 cm^−1^ are related to the hydroxy groups on the nanocomposite surface and water molecule. Also, the peaks that appeared in 1135.2 and 1382.4 cm^−1^ belong to the hydrogen bond between the boehmite plates^39^.

**Figure 3:**
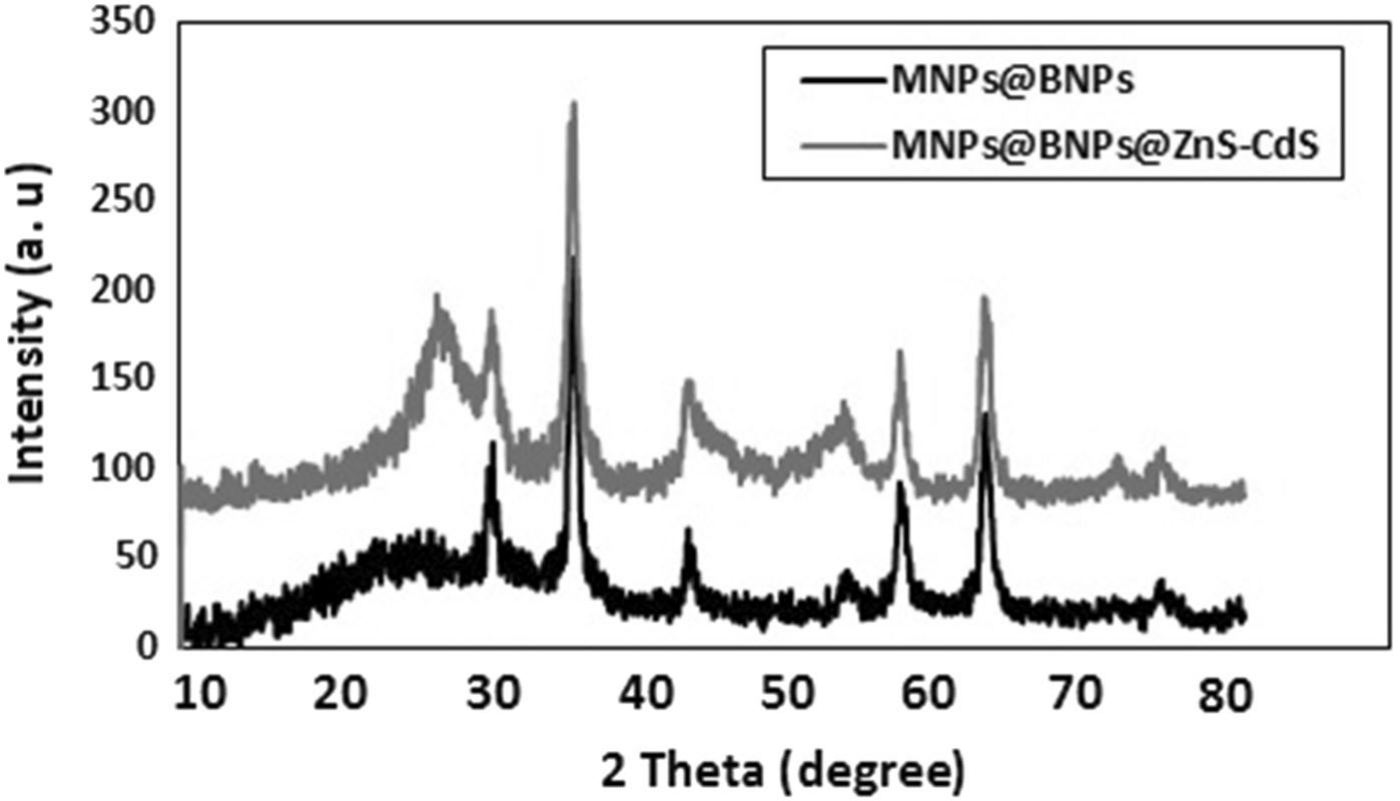
XRD diagrams of Fe_3_O_4_@BNPs and Fe_3_O_4_@BNPs@ZnS-CdS.

The XRD spectra of Fe_3_O_4_@BNPs and Fe_3_O_4_@ BNPs @ ZnS-CdS (0.25: 0.75) are shown in Fig. 3. In the XRD pattern of Fe_3_O_4_@MBPs, peaks appeared at 38.30, 35.79, 43.49, 53.92, 57.45 and 63.00 (01-075-0449 JCPDS No.), which have miller coefficients of (440), (511), (422), (400), (311), (220), respectively confirm the cubic structure of Fe_3_O_4_^31^. The peaks at 37.51 and 72.72 affirm the presence of BNPs in the structure.

In the XRD pattern of the Fe_3_O_4_@BNPs@ZnS-CdS, the peaks at 27.12 (111), (220) 45.62, and 64.01 are related to CdS (JCPDS No. 01-089-0440). The peaks seen at 57.03, 72.02 also show the presence of ZnS in the photocatalyst structure. (JCPDS No. 01-080-0020).

The other peaks which appear at 30.44, 35.72, 45.01.43, 43.38, 63.53 and 74.57, belong to the iron nanoparticles in the photocatalyst structure (JCPDS No. 01-075-0449). The single peak at 72.02 refers to BNPs. Of course, it should be noted that the peaks of boehmite nanoparticles overlap with the peaks of MNPs and CdS. The size of nanoparticles was calculated by well-known Debye Scherrer's formula as follows^40^:

1 $$ D = \, Kl/bCos\theta $$

Here, *D* is the crystallite size, *K* is the shape factor, calculated for spherical particles is 0.9, *λ* = 1.54 Å for Cu and *β* is full width at half maxima of the highest peak in radian. Based on the equation, the crystalline size for CdS was calculated between 6 and 37.9 nm, and the crystalline size for ZnS was between 26.7 and 29.1 nm.

**Figure 4:**
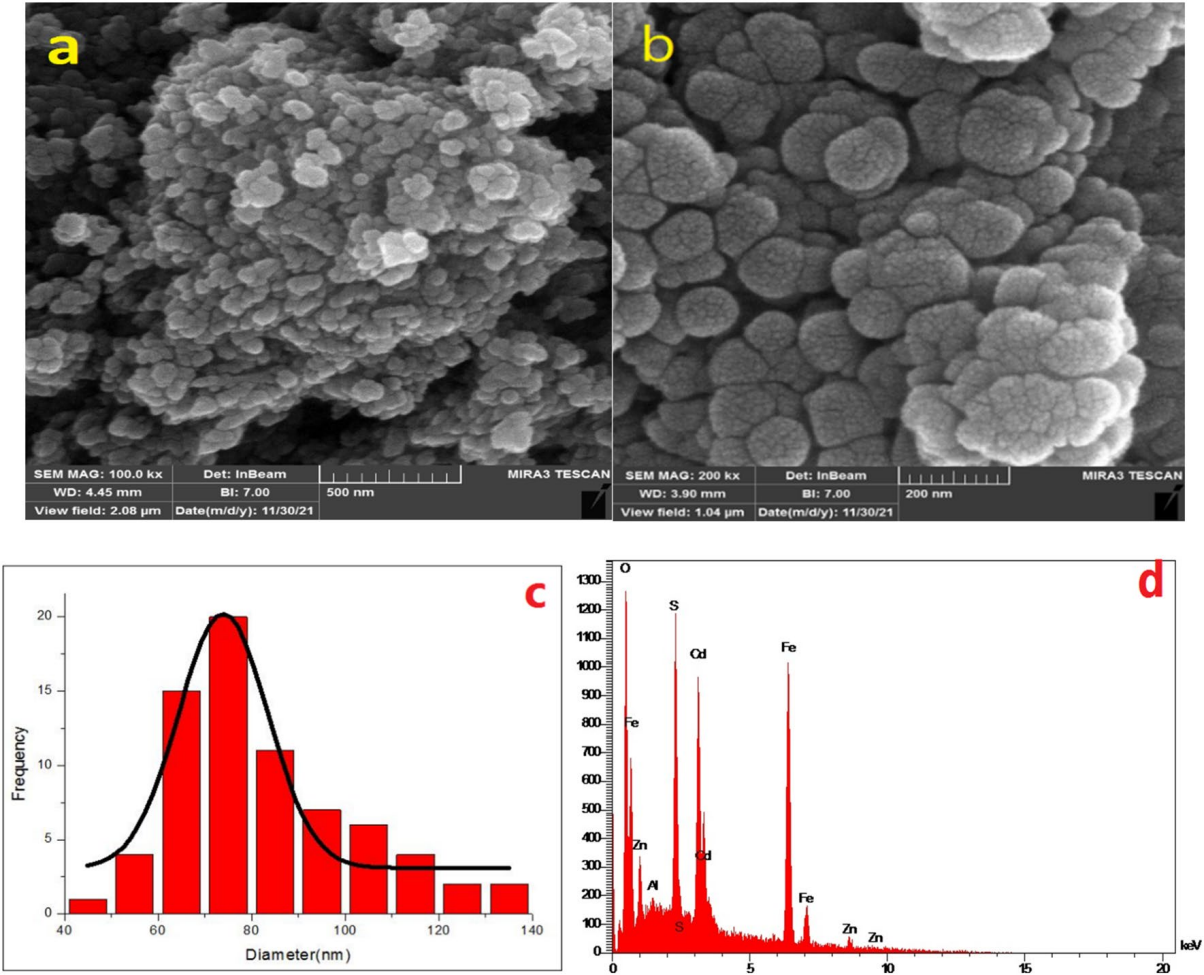
FESEM images of (**a**) Fe_3_O_4_@BNPs, (**b**) Fe_3_O_4_@BNPs@ZnS-CdS and (**c**) histogram size distribution of Fe_3_O_4_@BNPs@ZnS-CdS, and (**d**) EDX pattern of Fe_3_O_4_@BNPs@ZnS-CdS.

According to FESEM images, the morphology of magnetic boehmite is spherical and the distribution of particles is uniform (Fig. 4a). As can be seen from Fig. 4b, after rectification with cadmium sulfide- zinc sulfide, in addition to preserving the spherical structure of the nanoparticles, the nanoparticles were also evenly distributed on the surface of the magnetic boehmite.

The particle distribution histogram was used to determine the exact particle size and distribution of the particles. According to Fig. 4c, nanoparticles with a size between 60 and 100 nm have the highest frequency in the histogram of Fe_3_O_4_@BNPs@ZnS-CdS.

The chemical purity and elemental composition of the prepared Fe_3_O_4_@BNPs@ZnS-CdS was investigated by EDX technique. As Fig. 4d shows, all the major elements such as Al, Fe, O, S, Zn, Cd are present in the texture of the photocatalyst.

**Figure 5:**
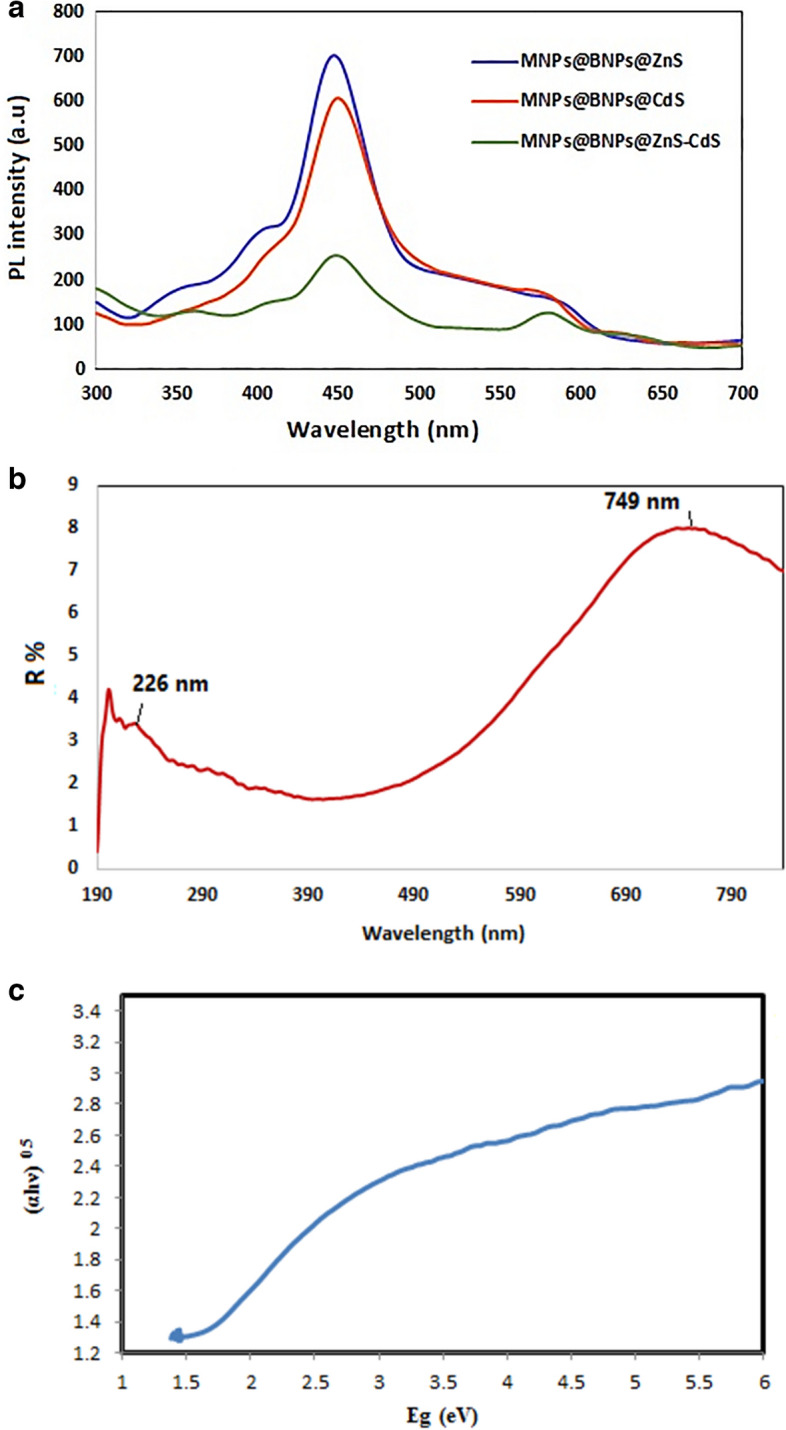
(**a**) PL spectra of Fe_3_O_4_@BNPs@ZnS-CdS, Fe_3_O_4_@BNPs@ ZnS and Fe_3_O_4_@BNPs@CdS (**b**) DRS spectrum of nanocomposite and (**c**) Tauc plots of the Fe_3_O_4_@BNPs@ZnS-CdS.

According to photoluminescence spectroscopy, the shorter the height of the emission spectrum, the lower the electron–hole recombination rate and the synthesized photocatalyst is more active. Figure 5a shows the photoluminescence spectra of the Fe_3_O_4_@ BNPs@ ZnS-CdS, Fe_3_O_4_@ BNPs @ZnS and Fe_3_O_4_@ BNPs-CdS photocatalysts. The emission spectrum of the Fe_3_O_4_@BNPs@ ZnS-CdS is the shortest of all, so the electron–hole recombination rate is the lowest for this photocatalyst.

DRS spectrum and band gap of the prepared Fe_3_O_4_@BNPs@ ZnS-CdS are shown in Fig. 5b, c, respectively. Band gap was calculated using Tauc plots. The intercept of the tangent to the plot of (αhν)^0.5^ versus (Eg) expresses the energy of the band gap with a good approximation. It is worth noting that the band gap for synthesized nanocomposites is much shorter (less than 2 eV) compared to the band gap for CdS and ZnS, which are 2.42 eV^41^ and 3.6 eV^42^, respectively.

**Figure 6:**
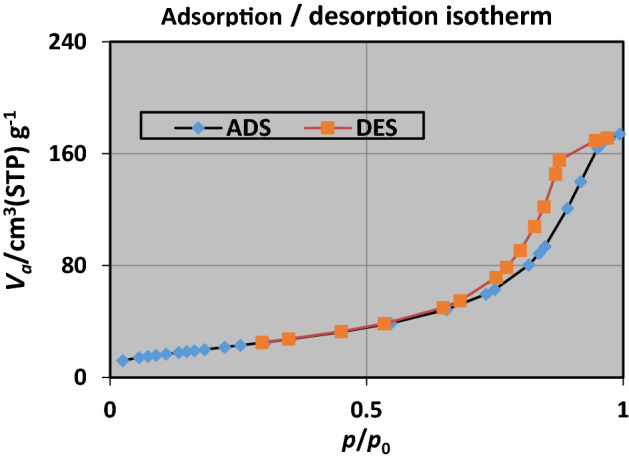
N_2_ absorption and desorption diagrams.

Based on BET test specific surface area (77.66 m^2^ g^−1^), particle volume (17.48 cm^3^ g^−1^), total pore volume (0.268 cm^3^ g^−1^) and mean pore diameter (13.83 nm) for optimal photocatalyst Fe_3_O_4_@ BNPs@ZnS-CdS (0.25/0.75) was obtained. Also, based on Langmuir test, particle volume (20.704 cm^3^ g^−1^) and specific surface area of 90.114 m^2^ g^−1^ were obtained. The nitrogen absorption and desorption diagrams (Fig. 6) confirm that the synthesized photocatalyst has a mesoporous structure and follows the type (IV) isotherm.

**Figure 7:**
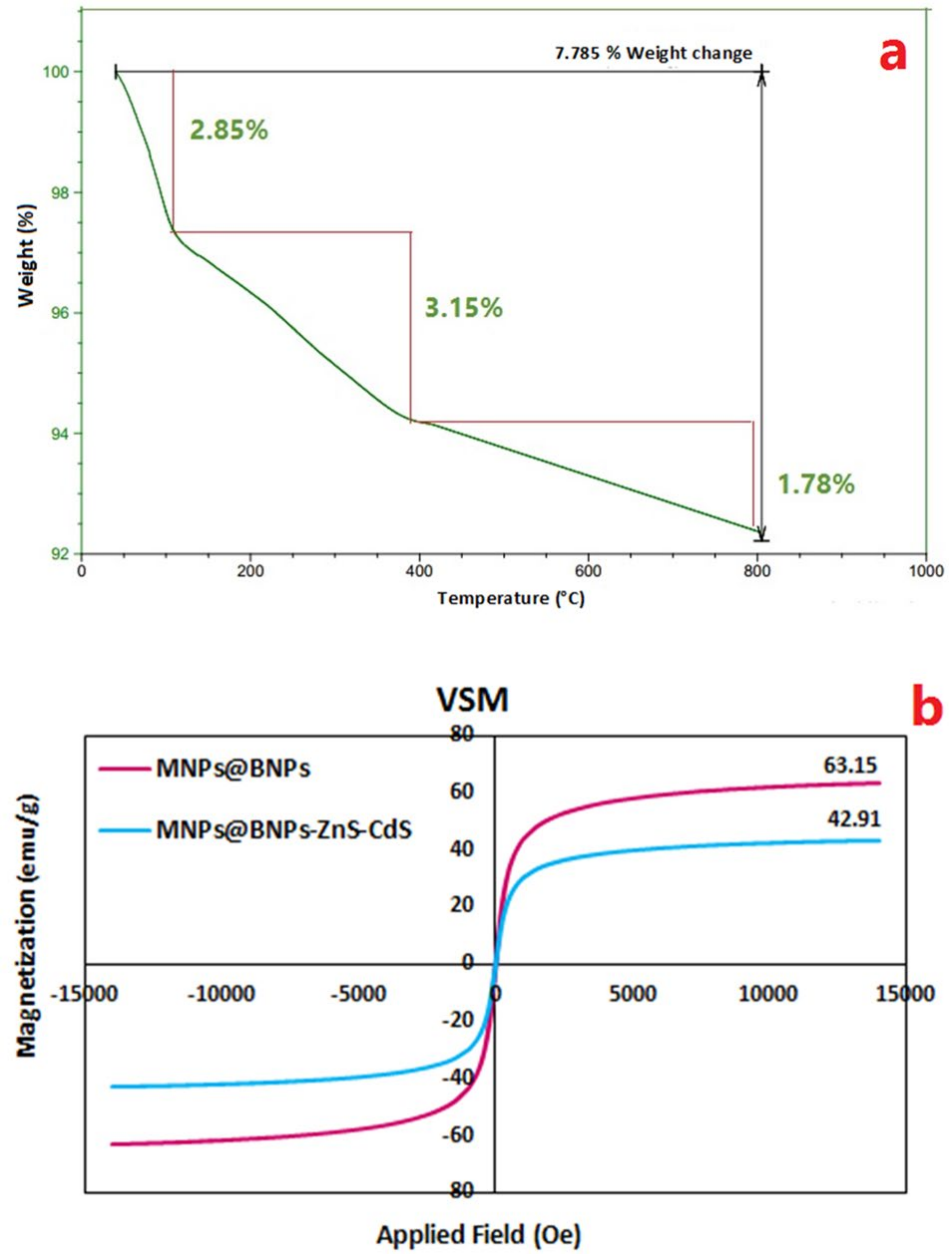
(**a**) TGA diagram for Fe_3_O_4_@BNPs@ZnS-CdS and, (**b**) VSM analysis for Fe_3_O_4_@BNPs@ZnS-CdS and Fe_3_O_4_@BNPs.

In total, 7.78% of the photocatalyst weight is lost during three failures (Fig. 7a). The first weight loss occurs in the range of 50–100 °C, which is related to adsorbed water and moisture. Subsequent weight loss (3.15%) observed in the range of 100–400 °C can be due to chemical transformations and physical changes such as the change of ZnS structure to wurtzite structure and separation of ZnS and CdS nanoparticles from the photocatalyst surface^43–45^.

The last loss occurs at 400–800 °C, which is related to the CdS separation and the boehmite crystal phase change^46^.

Figure 7b shows the magnetic strength of MNPs@BNPs and the final photocatalyst of Fe_3_O_4_@ BNPs@ZnS-CdS with a molar ratio of ZnS: CdS = 0.25:0.75. According to VSM analysis, the magnetic strength of MNPs@BNPs is 63.15 emu g^−1^ and the photocatalyst’s magnetic strength is 42.91 emu g^−1^. Despite surface modification with ZnS and CdS, the photocatalyst displays remarkable magnetic strength.

**Figure 8:**
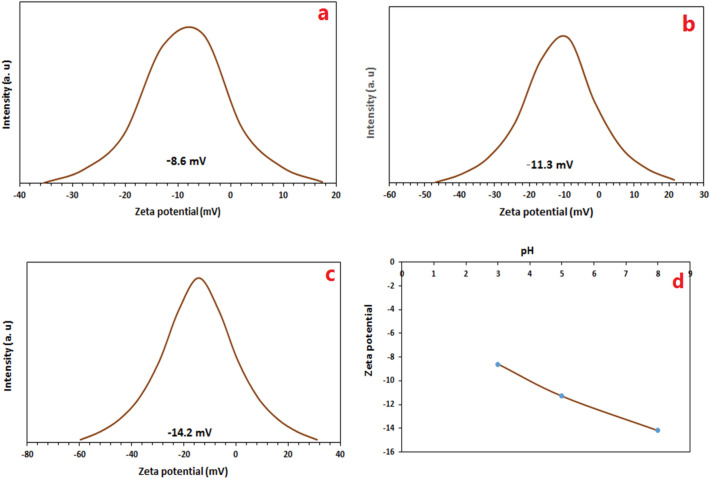
Gaussian distribution of charge for the Fe_3_O_4_@BNPs@ZnS-CdS at different pHs (**a**) pH = 3, (**b**) pH = 5, (**c**) pH = 8 and (**d**) Zeta potential versus pH curve for Fe_3_O_4_@BNPs@ZnS-CdS.

The dispersing stability of the photocatalyst nanoparticle is important during the dye removal process. In dye removal process, in order to perform better, the particles of the nanocatalyst surface should be dispersed and not aggregated. The dispersing stability can be predicted by measuring the zeta potential. Zeta potential shows the isoelectric point (the point where the net charge of the photocatalyst surface becomes zero). Of course, in some nanocomposites at all *p*Hs, only the surface charge is negative or positive and the isoelectric point is not observed. At the isoelectric point where the surface charge of the photocatalyst is zero, particles tend to accumulate, which reduces the performance of the photocatalyst^47,48^. Zeta potential was used to determine the surface charge of the photocatalyst in acidic, neutral and alkaline environments. The zeta potential curves for the photocatalyst at pHs 3, 5 and 8 are shown in Fig. 8, and the values for the zeta potential are shown in Table 1. Also, zeta potential curve against pH for the Fe_3_O_4_@BNPs@ZnS-CdS was illustrated in Fig. 8d. For the introduced photocatalyst, the charge on the surface of the photocatalyst is negative at all *p*Hs, and the particles repel each other and do not accumulate.

**Table 1:** Zeta potential at various pHs.

Entry	pH	Zeta potential (mv)
1	3	− 8.6
2	5	− 11.3
3	8	− 14.2

In this study, in addition to Fe_3_O_4_@BNPs @CdS and Fe_3_O_4_@BNPs@ZnS, three photocatalysts with different molar ratios of zinc sulfide-cadmium sulfide were synthesized and their photocatalytic performance in visible and ultraviolet light was investigated. After laboratory studies, it was found that the most efficient photocatalyst in the visible light region is Fe_3_O_4_@ BNPs@ZnS-CdS with a molar ratio of ZnS:CdS = 0.25:0.75, which has unique photocatalytic activity.

The studies were performed with aqueous solution of MB and MO with a concentration of 10 ppm in the presence of the mentioned photocatalysts (Table 2).

**Table 2:** Experiments to find the best photocatalyst in UV and visible regions.

Entry	Catalyst^a^	Dye	Light	Dye removal (%)	Time (min)
1	Fe_3_O_4_@BNPs-ZnS	MB	Visible	37	90
2	Fe_3_O_4_@BNPs-ZnS	MB	UV	25.4	90
3	Fe_3_O_4_@BNPs-ZnS	MO	Visible	33	90
4	Fe_3_O_4_@BNPs-ZnS	MO	UV	21	90
5	Fe_3_O_4_@BNPs-CdS	MB	Visible	74.2	90
6	Fe_3_O_4_@BNPs-CdS	MB	UV	10	90
7	Fe_3_O_4_@BNPs-CdS	MO	Visible	48	90
8	Fe_3_O_4_@BNPs-CdS	MO	UV	2.5	90
9	Fe_3_O_4_@BNPs@ZnS-CdS (0.25:0.75)	MB	Visible	96.6	90
10	Fe_3_O_4_@BNPs@ZnS-CdS (0.25:0.75)	MB	UV	47.6	90
11	Fe_3_O_4_@BNPs@ZnS-CdS (0.25:0.75)	MO	Visible	70.9	90
12	Fe_3_O_4_@BNPs@ZnS-CdS (0.25:0.75)	MO	UV	54.2	90
13	Fe_3_O_4_@BNPs@ZnS-CdS (0.5:0.5)	MB	Visible	76.2	90
14	Fe_3_O_4_@BNPs@ZnS-CdS (0.5:0.5)	MB	UV	65.2	90
15	Fe_3_O_4_@BNPs@ZnS-CdS (0.5:0.5)	MO	Visible	51	90
16	Fe_3_O_4_@BNPs@ZnS-CdS (0.5:0.5)	MO	UV	17	90
17	Fe_3_O_4_@BNPs@ZnS-CdS (0.75:0.25)	MB	Visible	51.7	90
18	Fe_3_O_4_@BNPs@ZnS-CdS (0.75:0.25)	MB	UV	45	90
19	Fe_3_O_4_@BNPs@ZnS-CdS (0.75:0.25)	MO	Visible	40	90
20	Fe_3_O_4_@BNPs@ZnS-CdS (0.75:0.25)	MO	UV	57	90

^a^gr of photocatalyst = 0.08 gr.

**Figure 9:**
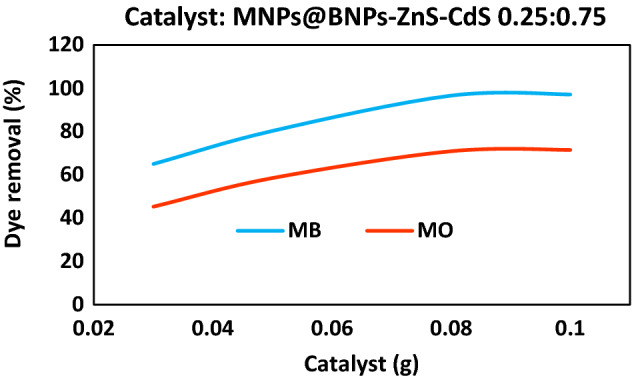
Effect of Fe_3_O_4_@BNPs-ZnS-CdS value on dye removal.

The effects of different dosages of Fe_3_O_4_@BNPs-ZnS-CdS for photocatalytic degradation in visible light region were explored (Fig. 9). Based on the results, 0.08 g was selected as the optimal amount of the photocatalyst. The use of higher amounts of photocatalyst had little (1%) effect on dye removal.

After selecting the light source and achieving the optimum amount of photocatalyst, dye elimination from synthetic MB and MO effluents was performed in the presence of high pressure mercury lamp as visible light source. First, the dye removal reactions for methylene blue and methyl orange were investigated in dark (in the absence of a high-pressure mercury lamp) in the presence of Fe_3_O_4_@BNPs-ZnS-CdS. After 1 h, about 5% and 1% of MB and MO dyes were removed, respectively. Also, the effect of photolysis was studied so that the dye removal reactions of MB and MO in the absence of photocatalyst were exposed to high pressure mercury lamp radiation for 1 h. The amount of photolysis was 2% for MO and 3% for MB.

**Figure 10:**
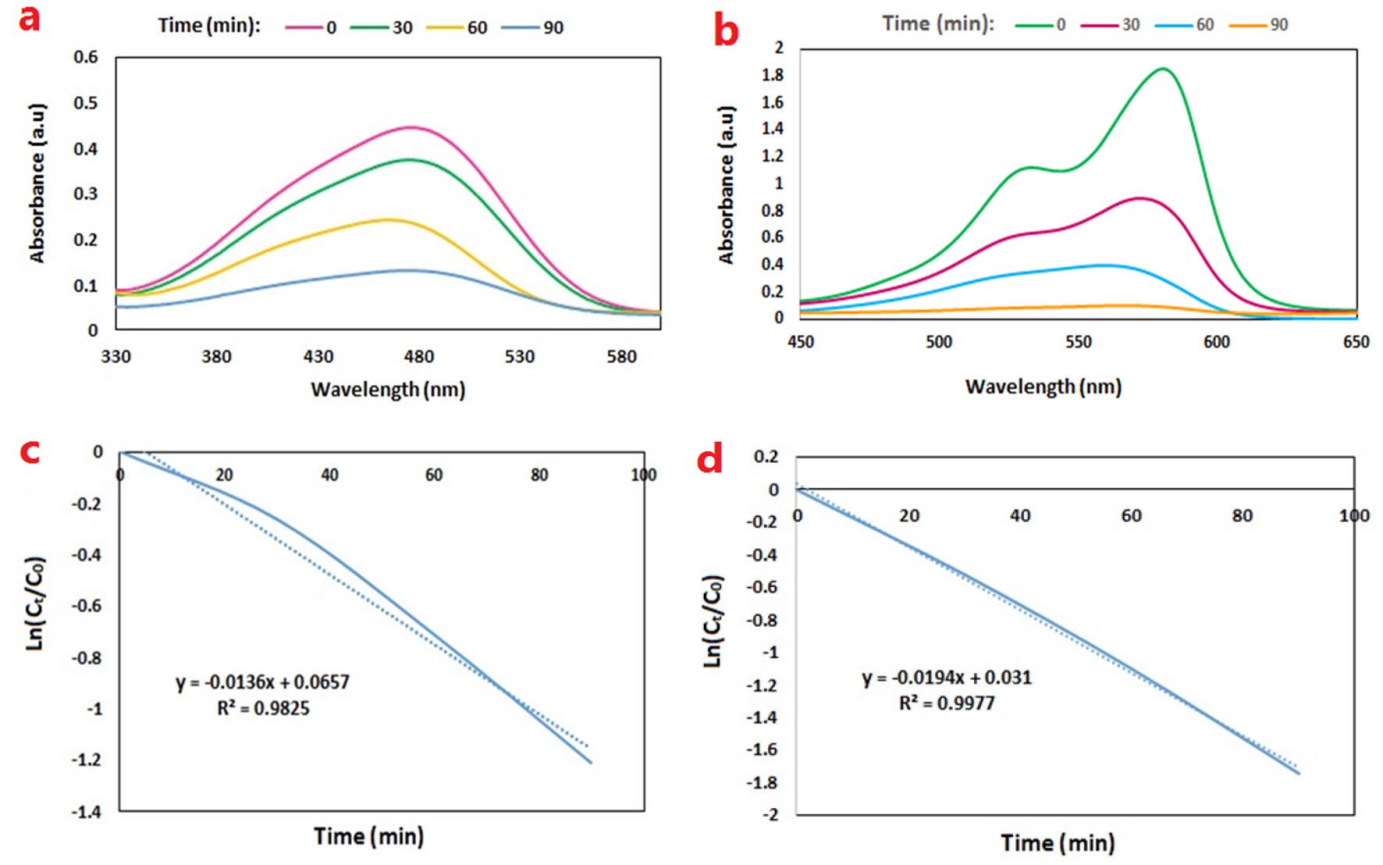
Photocatalytic removal of (**a**) MO and (**b**) MB in the presence of Fe_3_O_4_@BNPs-ZnS-CdS and pseudo-first-order kinetic curves for (**c**) MO and (**d**) MB.

The amount of dye removal in the presence of Fe_3_O_4_@BNPs-ZnS-CdS (0.25: 0.75) for 10 ppm MB solution was 96.6% and for 10 ppm MO solution was 70.9% (Fig. 10a, b). In all studies, the amount of photocatalyst is 0.08 gr and the irradiation time is 90 min. In order to achieve the dye removal efficiency, after the appropriate time, the photocatalyst was removed from the environment. Then, degradation of MB and MO were monitored by a UV–Vis spectrophotometer at λ max for each dye. The maximum absorption band is 470 nm for MO and 580 nm for MB.

Dye removal efficiency was calculated using the following equation:

2 $$ {\text{Removal }}\left( \% \right) \, = \, \left( {{\text{A}}_{0} - {\text{A}}_{{\text{t}}} /{\text{A}}_{0} } \right) \, \times { 1}00 $$

where A_0_ is the adsorption of dye solution at time = 0 and A_t_ is the adsorption of the final sample at time t.

Pseudo-first-order kinetics were obtained for dye elimination after calculations. The rate constant was calculated from the following equation:

3 $$ {\text{Ln }}\left( {{\text{A}}_{{\text{t}}} /{\text{A}}_{0} } \right) \, = {\text{ ln}}(C_{t} /C_{0} ) \, = \, - k_{{{\text{app}}}} t $$

In this equation, C_0_ is the organic dye concentration at t = 0 and C_T_ is the organic dye concentration at time t.

The kinetic graphs and K_ap_ (removal rate constant) for the photocatalytic decolorization of MO and MB are shown in Fig. 10c, d. Based on the calculations, K_ap_ for MO dye (0.0136 min^−1^) and for MB dye (0.0194 min^−1^) were obtained and the photocatalytic elimination of MO and MB dyes follows the pseudo-first-order rate constant.

The reproducibility of dye removal reactions for synthetic effluents (MB and MO) was also investigated under optimal conditions up to four times in a row. As expected, dye removal reactions showed excellent reproducibility for both synthetic effluents. Dye removal efficiencies for methyl orange and methylene blue were 96.6% and 70.9% in all four times, respectively.

Based on the results of the zeta potential test, the surface of the photocatalyst has a negative charge, so cationic dyes such as methylene blue are more easily attracted to the surface of the photocatalyst based on electrostatic attraction and are destroyed more efficiently. Anionic dyes such as methyl orange are less absorbed on the surface due to electrostatic repulsion and the amount of dye degradation on the photocatalyst surface is lower.

The effect of increasing dye concentration on dye removal rate for MO and MB dyes in the presence of Fe_3_O_4_@BNPs-ZnS-CdS photocatalyst under visible light was checked. For this purpose, concentrations (10, 15 and 20 ppm) of the mentioned dyes were studied and the results are summarized in Table 3. As the table demonstrates, increasing the concentration of the dye solution does not have a significant effect on the percentage of dye elimination.

**Table 3:** Effect of dye’s concentration on photocatalytic decolorization.

Entry	Dye	Concentration (ppm)	Dye removal (%)^a^	Light
1	MO	10	70.9	Visible
2	MO	15	70.9	Visible
3	MO	20	70.3	Visible
4	MB	10	96.6	Visible
5	MB	15	95.5	Visible
6	MB	20	95.2	Visible

^a^Optimal catalyst (Fe_3_O_4_@BNPs-ZnS-CdS with ZnS/CdS molar ratio: 0.75/ 0.25).

**Figure 11:**
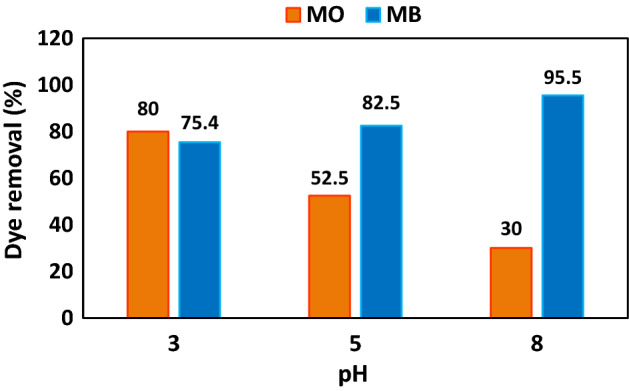
pH effect on the photocatalytic dye removal of MO and MB dyes.

Photocatalytic dye elimination of MO and MB dyes was surveyed with optimized Fe_3_O_4_ @ BNPs-ZnS-CdS photocatalyst under visible light at various pHs (3, 5 and 8). According to Fig. 11, the MO dye removal percent is lower than the neutral medium (70.9%) at all pHs except pH 3. As the diagram illustrates, in the case of MB, the dye elimination in neutral medium (96.6%) is higher than in acidic and alkaline media. The obtained results can be interpreted by the surface charge of the photocatalyst and the nature of the dye. Usually, in photocatalytic processes, dyes are first absorbed on the surface, and then dye degradation occurs on the photocatalyst surface^49^.

Figure 11 shows that methyl orange, which is an anionic dye, has a higher degradation rate at pH 3, where the surface charge of the photocatalyst is the smallest compared to pH 5 and 8. At pH 3, because the amount of negative charge on the photocatalyst surface is less, the amount of electrostatic repulsion of the photocatalyst surface with the dye molecule (MO) is the lowest, so the dye degradation rate is higher. These results are completely consistent with the results obtained from zeta potential. As for methylene blue, since it is a cationic dye with a negative charge on the surface of the photocatalyst, it has electrostatic attraction. The higher the negative charge, the higher the dye absorption and dye degradation on the photocatalyst surface. As can be seen from Fig. 10, the performance of the photocatalyst at pH 8, which has the highest amount of negative charge, is higher at pH 3 and 5.

**Figure 12:**
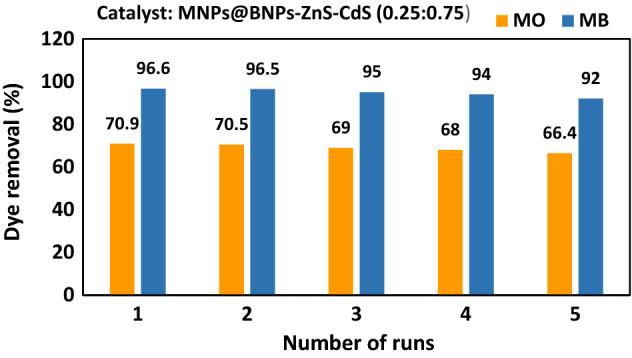
The renewability of Fe_3_O_4_@BNPs@ZnS-CdS for elimination of MB and MO.

It is noteworthy that the retrievability of photocatalyst Fe_3_O_4_@BNPs@ZnS-CdS (0.25: 0.75) for MO and MB (10 ppm) dyes under visible light up to five times was investigated. About 5% decrease in photocatalytic activity was observed after five sequential uses (Fig. 12).

Table 4 compares the photocatalytic performance of synthesized magnetic nanocomposites with some other photocatalytic systems. As can be deduced from the table, the hybrid photocatalyst (Fe_3_O_4_@BNPs@ZnS-CdS) is superior to most reported photocatalysts in terms such as dye degradation time and dye removal rate. Also in this study, high pressure mercury lamps were used as a cheap, durable and efficient light source.

**Table 4:** Compration of photocatalytic performance of Fe_3_O_4_@BNPs@ZnS-CdS with some photocatalytic systems.

Catalyst	Light sources	Dyes	Degradation	Refs
ZnS/CdS/Ag_2_S	Sun light	Congo red	97%, 120 min	^50^
Co_0.5_Zn_0.25_Cu_0.25_Fe_2_O_4_-TiO_2_	Solar	MO	50%, 360 min	^51^
Cs-ZnS-NPs	Uv	Acid brown	92%, 180 min	^52^
N, S and Zn doped TiO_2_	Visible light	MB	96%, 35 min	^53^
Fe_3_O_4_@SiO_2_@ZnO-ZnS	Visible light	MB	92%, 180 min	^54^
CdS/ZnS	Visible light	MB	70%, 360 min	^55^
CdS/TiO_2_	Visible light	MB	60%, 180 min	^56^
Co@C-N-S triple doped TiO_2_	Visible light	MO	90%, 360 min	^57^
L-cysteine (2%) doped TiO_2_/CdS (10%)	Visible light	MB	93%, 180 min	^25^
ZnS-Cds-PANI	Visible light	MB	45%, 60 min	^58^
ZnO-ZnS	Visible light	MB	95%, 135 min	^59^
ZnO + Alginate 2%	Uv	MB	63%, 240 min	^60^
ZnO-ZnS-MnO_2_	Visible light	MB	97%, 140 min	^40^
ZnS-CdS	Uv–visible	MO	44.1%. 120 min	^61^
Fe_3_O_4_@BNPs@ZnS-CdS	Visible light	MB	96.5%, 90 min	This work

### Probable mechanism and active radical species in dye removal

**Figure 13:**
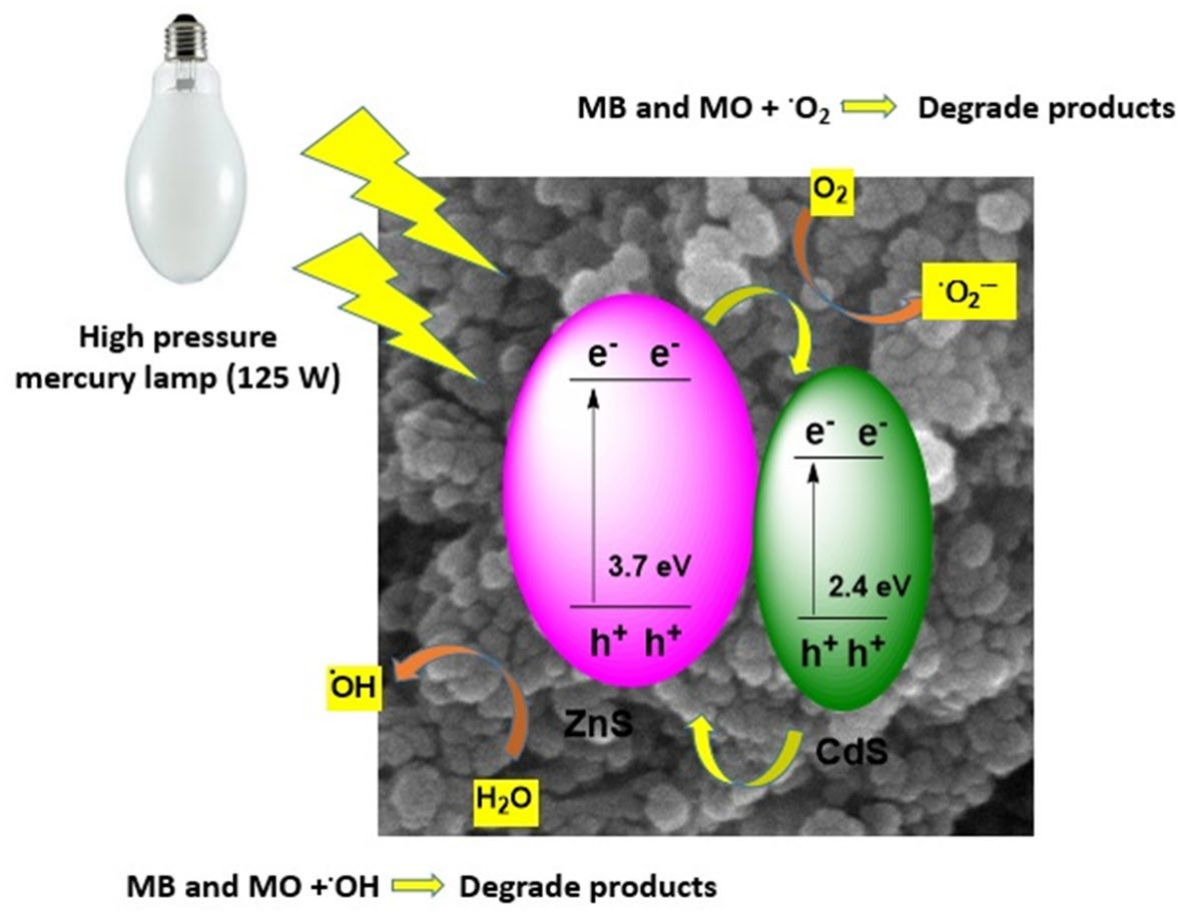
Schematic illustration of dye degradation over Fe_3_O_4_@BNPs@ZnS-CdS.

Photocatalytic degradation processes for MO and MB are illustrated Eqs. (4–8) and Fig. 13.

4 $$ {\text{ZnS }} + {\text{ h}}\upsilon \, \to {\text{ h}}^{ + } + {\text{ e}}^{ - } \;{\text{and}}\;{\text{ CdS }} + {\text{ h}}\upsilon \, \to {\text{ h}}^{ + } + {\text{ e}}^{ - } $$

The oxidative and reductive reactions are expressed as:

5 $$ {\text{OH}}^{{ - }{}} + {\text{ h}}^{ + } \to {}^{ \cdot }{\text{OH}} $$

6 $$ {\text{H}}_{{2}} {\text{O }} + {\text{ h}}^{ + } \to {}^{ \cdot }{\text{OH }} + {\text{ H}}^{ + } $$

7 $$ {\text{O}}_{{2}} + {\text{ e}}^{ - } \to {\text{ O}}_{{2}}^{ \cdot - } + {\text{ H}}^{ + } \to {\text{ HO}}_{{2}}^{\cdot} $$

Hydroxyl radicals (^**·**^OH) are obtained from the oxidation of absorbed water or absorbed hydroxyl anion (OH^-^). Also, the presence of oxygen prevents electron–hole recombination. Under the photocatalytic process, the dyes are converted into decomposition products in the presence of hydroxyl radicals and eventually turn into water and carbon dioxide^62–64^.

8 $$ {\text{Dye }}\left( {\text{MO or MB}} \right) + {}^{ \cdot }{\text{OH or O}}_{{2}}^{ \cdot - } \to {\text{ Degrade }}\;{\text{products }}\left( {{\text{CO}}_{{2}} + {\text{ H}}_{{2}} {\text{O }}\;{\text{and }}\;{\text{other }}\;{\text{products}}} \right) $$

**Figure 14:**
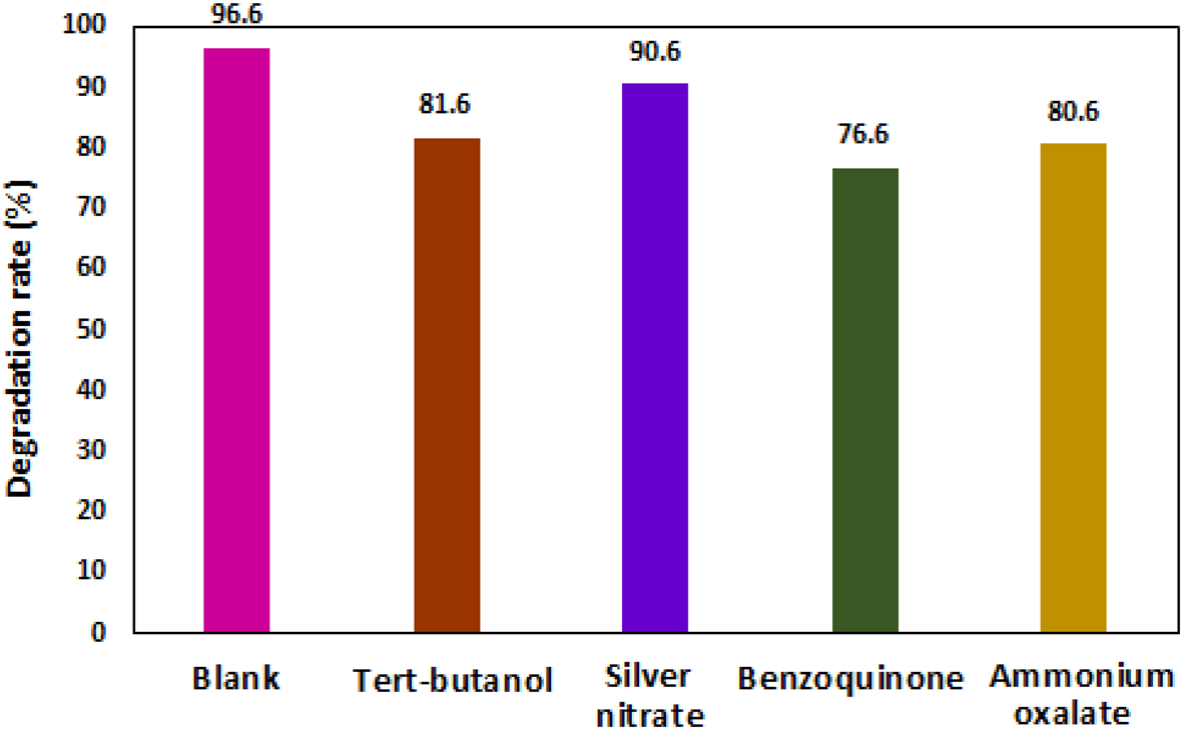
Effect of various scavengers on the photocatalytic degradation of MB in the presence of high-pressure mercury lamp.

In order to show active radical species in the photocatalytic process of MB removal, several radical scavengers such as benzoquinone, ammonium oxalate, silver nitrate and tert-butanol were used (Fig. 14). Active species responsible for dye degradation in the presence of photocatalyst are (^**·**^OH), superoxide anion radical (O_2_^**·**−^), e^−^ and h^+^^65,66^. About 96.6% of methylene blue was removed in the absence of quencher under optimal conditions within 90 min. In this study, benzoquinone, ammonium oxalate, Ag(NO_3_) and tert-butanol were used as scavengers of O_2_^**·−**^, h+, e^−^ and ^**·**^OH, respectively. Dye degradation was decreased about 20% when benzoquinone was added as O_2_^**·−**^ quencher. After using ammonium oxalate, which acts as h^+^ scavenger, the removal of methylene blue decreased by 16%. About 6% reduction in the degradation of MB was observed when using silver nitrate, which indicates that electrons do not play a key role in the photocatalytic degradation of methylene blue.

In the presence of tert- butanol (used as a scavenger to quench OH), the degradation rate of MB declined by 15%^49^. Based on the results of O_2_^**·−**^, h+, and ^**·**^OH species are the main species responsible for the photocatalytic degradation of dyes. Based on the results, each scavenger had a different effect on the photocatalytic MB degradation. Also, based on Eqs. (6) and (7), apart from O_2_^−^, h^+^, and OH^**·**^ mentioned above, other active species such as H^+^, HO_2_^**·**^, … are created on the surface of the photocatalyst, which can be effective in dye degradation.

Considering that cadmium sulfide is toxic and its release into water and environment is dangerous for the health of living organisms and humans, the issue of Cd^+2^ leaching from the photocatalyst surface into the solution was investigated. For this purpose, at the end of the photocatalytic dye removal process, about 1 mL of final solution was reacted with sodium sulfide under stirrer. Since cadmium sulfide is insoluble in water, adding sodium sulfide to any solution containing a small amount of cadmium should produce a yellow precipitate of cadmium sulfide. From the point of view of chemistry, the formation of cadmium sulfide precipitate is fast^67,68^.

### Efficiency of Fe_3_O_4_@BNPs@ZnS-CdS in photocatalytic dye removal of real effluents

In order to evaluate the efficiency of the photocatalyst, dye removal from textile, west carton wastewaters in the presence of visible light and in the absence of catalyst for 30–90 min were investigated. After the desired time, the amount of photolysis was 3% and 2%, respectively. Also, in order to determine the amount of dye absorption by the photocatalyst surface, dye removal of textile and carton effluents were evaluated in the presence of photocatalyst (0.08 gr) and in the absence of visible light at rt for 90 min. After that, the wastewater solutions were examined by UV–visible spectrophotometer and the percentage of dye adsorbed on the photocatalyst surface was 2 and 4, respectively.

The dye removal time for the actual effluent varied from 30 to 90 min and the amount of photocatalyst in all experiments was 0.08 g. In all studies, the reactor is placed in a container containing cold water to keep the temperature constant. Concentrations of 100, 50, 25, 10 ppm of carton effluent were examined using Fe_3_O_4_@BNPs@ ZnS-CdS at rt in the presence of high-pressure mercury lamps, which showed 89%, 92.5%, 97.1% and 100% dye removal, respectively.

Three textile effluents (pastel pink, benton and dark olive dyes, with concentrations of 10–20 ppm) in the presence of Fe_3_O_4_@ BNPs @ZnS-CdS (0.25: 0.75) were evaluated at rt.

**Figure 15:**
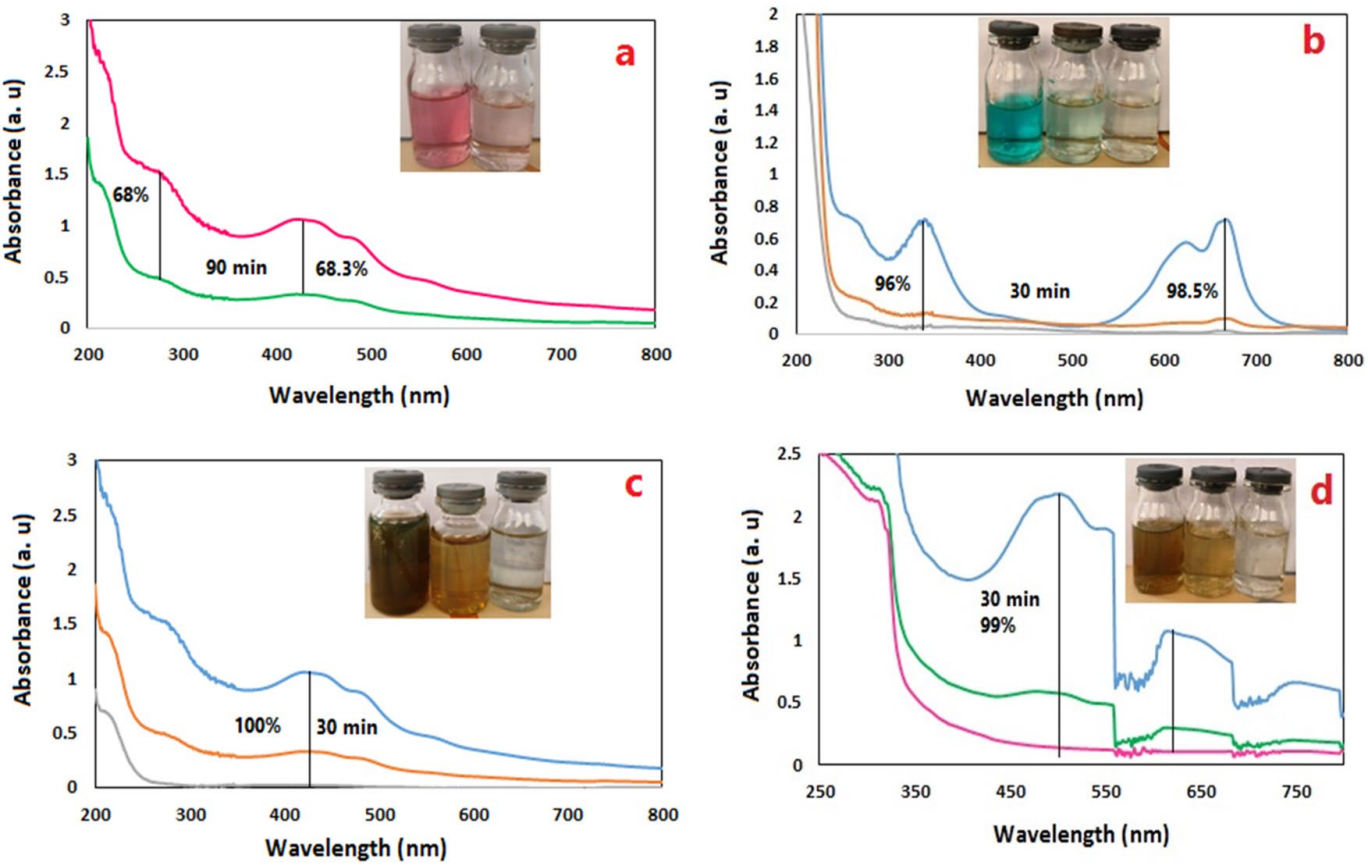
UV–visible spectra for (**a**) pastel pink (15 ppm), (**b**) benton (15 ppm), (**c**) carton wastewater (50 ppm), (**d**) dark olive (20 ppm).

Benton and dark olive effluents were completely bleached in 30 min with 100% efficiency. But pastel pink effluent showed about 68.3% percent of dye removal in 90 min. Kermanshah carton effluent with a concentration of 20 ppm also showed complete color removal in the presence of Fe_3_O_4_@ BNPs@ ZnS-CdS (0.25: 0.75) (Fig. 15a–d).

## Conclusion

Photocatalysts are favorable options for removing organic pollutants, especially dyes from water and wastewater. In this study, some magnetic composite photocatalysts were synthesized and their photocatalytic performance in dye elimination from synthetic and real effluents was investigated. In this survey, Fe_3_O_4_@BNPs@ZnS-CdS (0.25:0.75) is the best photocatalyst, which was identified by techniques such as FTIR, XRD, FESEM, EDX, TGA, DRS, DLS, PL, VSM, BET, and N_2_-adsorption desorption. The synthesized Fe_3_O_4_@BNPs@ZnS-CdS is stable, recyclable and highly efficient for removing dye from synthetic (MB: 96.6%) and real effluents (68- 100%) in the visible area.

Spherical structure and uniform arrangement of Fe_3_O_4_@BNPs@ ZnS-CdS nanoparticles were confirmed by FESEM. Stabilization of ZnS-CdS on the surface of magnetic nanocomposites was confirmed based on IR and XRD spectra. Using Tauc plot, the gap for the photocatalyst was calculated to be less than 2. Charge on the photocatalyst surface is negative at all pHs and therefore the synthesized photocatalyst is more efficient at removing cationic dyes than anionic dyes. Some advantages of this method include: use of relatively cheap materials, easy synthesis method, photocatalyst thermal stability, recyclability of photocatalyst, reproducibility of dye removal results, easy separation of photocatalyst by external magnet, use of cheap and durable lamps. In this study, high-pressure mercury lamps were used as the visible light source, which are very cheap, durable and efficient compared to other visible light lamps on the market.

## Data Availability

The data that support the findings of this study are available from the corresponding author upon reasonable request.
